# Synthetic bone marrow images augment real samples in developing acute myeloid leukemia microscopy classification models

**DOI:** 10.1038/s41746-025-01563-9

**Published:** 2025-03-22

**Authors:** Jan-Niklas Eckardt, Ishan Srivastava, Zizhe Wang, Susann Winter, Tim Schmittmann, Sebastian Riechert, Miriam Eva Helena Gediga, Anas Shekh Sulaiman, Martin M. K. Schneider, Freya Schulze, Christian Thiede, Katja Sockel, Frank Kroschinsky, Christoph Röllig, Martin Bornhäuser, Karsten Wendt, Jan Moritz Middeke

**Affiliations:** 1 Department of Internal Medicine I, University Hospital Carl Gustav Carus, TUD Dresden University of Technology, Dresden, Germany; 2 Else Kröner Fresenius Center for Digital Health, TUD Dresden University of Technology, Dresden, Germany; 3 Chair of Software Technology, TUD Dresden University of Technology, Dresden, Germany; 4 German Cancer Consortium (DKTK), Partner Site Dresden, and German Cancer Research Center (DKFZ), Heidelberg, Germany; 5 National Center for Tumor Diseases Dresden (NCT/UCC), Dresden, Germany

**Keywords:** Acute myeloid leukaemia, Acute myeloid leukaemia

## Abstract

High-quality image data is essential for training deep learning (DL) classifiers, yet data sharing is often limited by privacy concerns. We hypothesized that generative adversarial networks (GANs) could synthesize bone marrow smear (BMS) images suitable for classifier training. BMS from 1251 patients with acute myeloid leukemia (AML), 51 patients with acute promyelocytic leukemia (APL), and 236 stem cell donors were digitized, and synthetic images were generated using StyleGAN2-Ada. In a blinded visual Turing test, eight hematologists achieved 63% accuracy in identifying synthetic images, confirming high image quality. DL classifiers trained on real data achieved AUROCs of 0.99 across AML, APL, and donor classifications, with performance remaining above 0.95 even when incrementally substituting real data for synthetic samples. Adding synthetic data to real training data offered performance gains for an exceptionally rare disease (APL). Our study demonstrates the usability of synthetic BMS data for training highly accurate image classifiers in microscopy.

## Introduction

The term “big data” has become a buzzword in medical literature, yet its interpretation varies significantly between medical professionals and data scientists. For instance, ImageNet^1^ (https://www.image-net.org/)—an object-based image database frequently used for training and validation of computer vision models—currently contains over 14 million images. In contrast, medical image databases are often considered “big data” when patient samples range in the hundreds. While medical image data sets that reach sample sizes comparable to ImageNet are most likely unattainable (and limited by the prevalence of the disease of interest), increasing the size of publicly available data sets is still of utmost importance for the training and validation of computer vision models in medicine^2^. Since model robustness scales with training set size and overall data quality^3^, increasing the amount of training data is vital in cancer diagnostics, where accurate detection is a prerequisite for correct treatment decisions and errors can lead to life-threatening consequences. Given the rarity of hematological malignancies, training image-based classifiers is often cumbersome due to a lack of publicly available large data sets. Moreover, in contrast to radiology and histopathology, where public image repositories like The Cancer Imaging Archive (https://www.cancerimagingarchive.net/) hold a respectable amount of data sets, hematology still lacks sufficient digitization, resulting in a scarcity of publicly available images. Conventional methods for image augmentation such as geometric or photometric transformations are used to inflate the size of the training data^4^. Generative adversarial networks (GANs), introduced by Goodfellow et al.^5^, offer a more sophisticated method for sample size augmentation. GANs consist of a generator, which creates synthetic data mimicking a given data set, and a discriminator, which differentiates between real and synthetic samples. Ultimately, the network converges when the generator produces synthetic data that the discriminator can no longer distinguish from real data^5,6^.

We have recently demonstrated that convolutional neural networks (CNN) show excellent performance in detecting both acute myeloid leukemia (AML) and acute promyelocytic leukemia (APL)^7,8^. This study aims to:

1. generate a large quantity of synthetic AML and APL samples for public availability,
2. demonstrate synthetic image quality via a visual Turing test, in which hematologists are tasked with distinguishing between synthetic and real images, and
3. use synthetic samples to train image-based classifiers for AML, APL, and stem cell donor samples to demonstrate the feasibility of synthetic image data in training and validating computer vision models for bone marrow cytomorphology.

## Results

### GANs create realistic bone marrow images that are indistinguishable in visual Turing tests

**Fig. 1:**
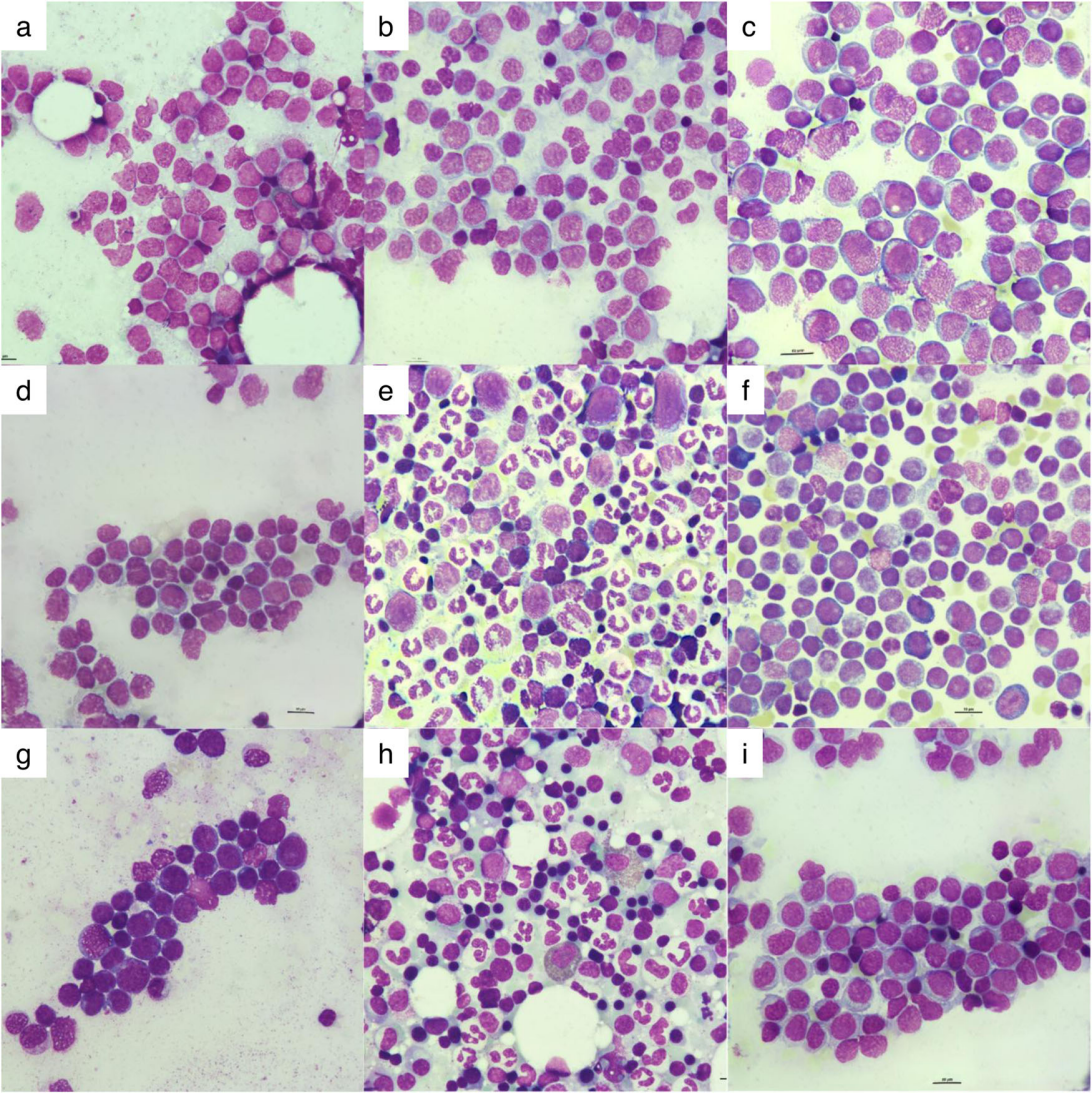
Example images used in the visual Turing test. Using a web application, eight experienced hematologists had to evaluate whether an image was real or synthetic. Images were presented one at a time and shuffled randomly. Evaluation was performed without time constraints. Real samples are shown in **a**, **f**, **g**, **h**, whereas **b**, **c**, **d**, **e**, and **i** are synthetic images.

First, we trained a StyleGAN2-Ada network to generate bone marrow image data for the two different leukemia cohorts comprising (1) 1251 non-APL AML patients and (2) 51 APL patients. We found that the network was able to generate high-quality images after processing 4000 training images (original sample size was augmented using geometric or photometric alterations). Examples of both real and synthetic images generated with the final model are shown in Fig. 1. For all real-synthetic image pairs, Euclidean distance similarity scores were used to evaluate how closely synthetic images resemble real images. A lower Euclidean distance indicates greater similarity between a real and synthetic image pair, whereas a higher Euclidean distance reflects greater dissimilarity. Median scores were 4.43 (IQR: 3.79–5.03; range 2.17–9.61) for AML, 4.95 (IQR: 4.35–5.72; range 2.46–11.01) for APL, and 3.98 (IQR: 3.62–4.40; range 2.28–7.29) for donors, indicating sufficient similarities between real-synthetic image pairs while avoiding direct replication (Supplementary Figs. 1–3). Further, pixel-wise comparisons via SSIM were calculated for six randomly selected synthetic images paired with all real images per use case. Median SSIM values were 0.26 for AML, 0.24 for APL, and 0.09 for donor images, demonstrating low structural similarity and indicating that synthetic images are unique instead of copying or modulating image elements from real images. Given that SSIM is very computationally expensive, it is not feasible to compute SSIM for all synthetic-real pairs. Supplementary Fig. 4 shows an example for each use case, where a synthetic image is compared to paired real images that had the highest SSIM scores, demonstrating that the synthetic images are clearly distinct from the real images. This further underscores the generative model’s ability to mimic realistic image features.

**Fig. 2:**
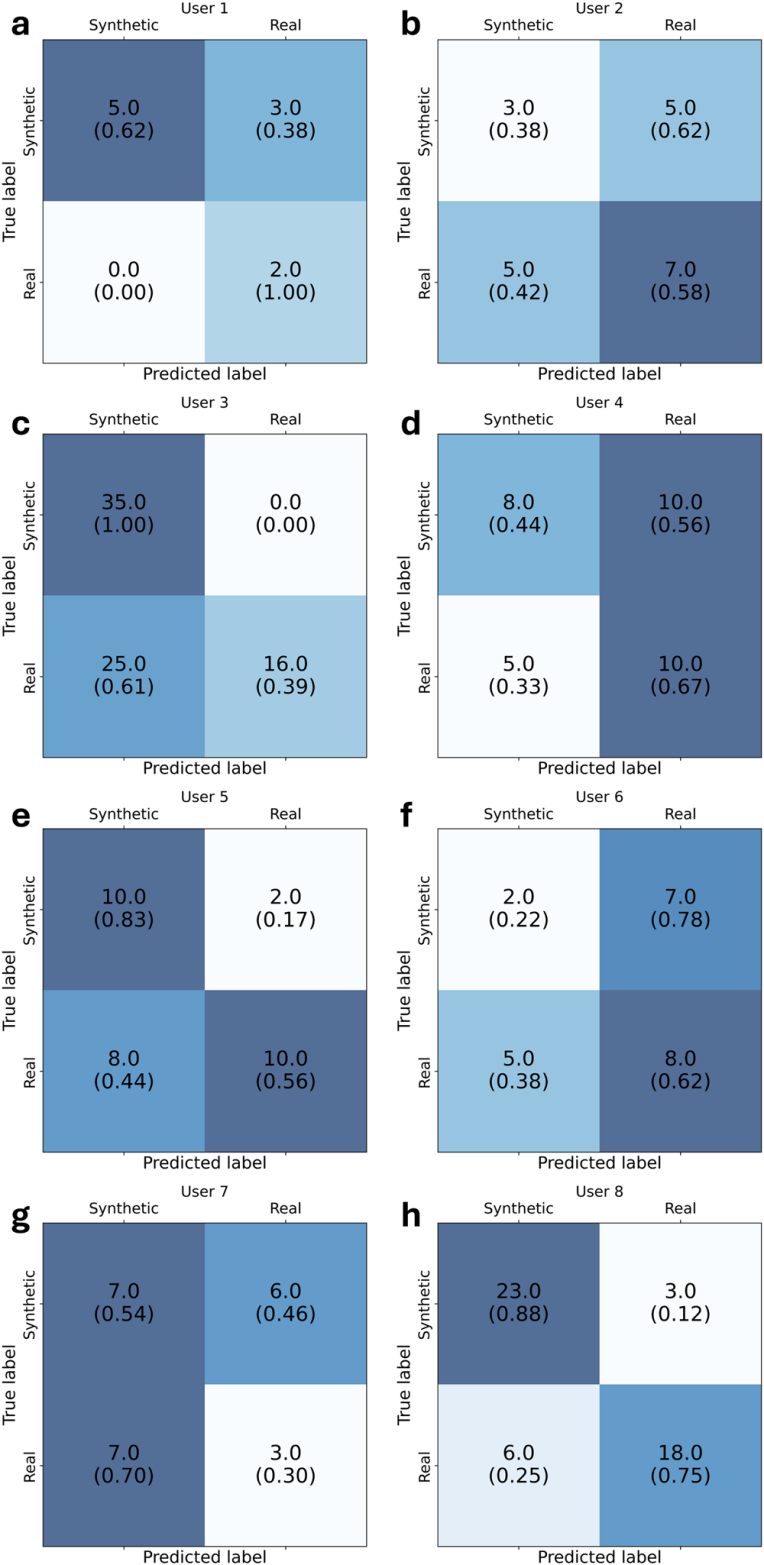
Visual Turing test results per user. In a web application, eight hematologists (**a**–**h**) were presented with one image at a time and tasked with evaluating whether they thought it was real or synthetic. The overall accuracy for detecting synthetic images was 63.26%. Confusion matrices show the number of images correctly and incorrectly classified per individual user. Numbers in brackets show the proportion of correctly and incorrectly classified images for each actual class. Notably, only user 3 (**c**) was able to reliably identify synthetic images, which he attributed to irregular chromatin structures in the generated images.

Furthermore, eight experienced hematologists evaluated the images via the web tool (Fig. 2a–h). Human observers had an average accuracy of 63.26% (95%-CI: 57.13% to 69.08%). They correctly identified 72.09% (95%-CI: 63.52% to 79.63%) of synthetic images as synthetic (sensitivity) and 54.81% (95%-CI: 46.02% to 63.39%) of real images as real (specificity), corresponding to a positive predictive value of 60.39% (95%-CI: 55.16% to 65.39%) and a negative predictive value of 67.27% (95%-CI: 59.96% to 73.83%). While most users reported notable difficulties in distinguishing synthetic and real images, and often overestimated the number of synthetic images (i.e. mistaking real images for synthetic images), user 3 had an outstanding accuracy in detecting synthetic images, which he attributed to irregularities in chromatin density especially in myeloblasts, reminiscent of a fabric-like texture in synthetic images. Nevertheless, while user 3’s accuracy in spotting synthetic images was remarkable, he as well as the other users often mislabeled real images as synthetic.

### Synthetic images are a viable alternative to real patient samples in classifier training

**Fig. 3:**
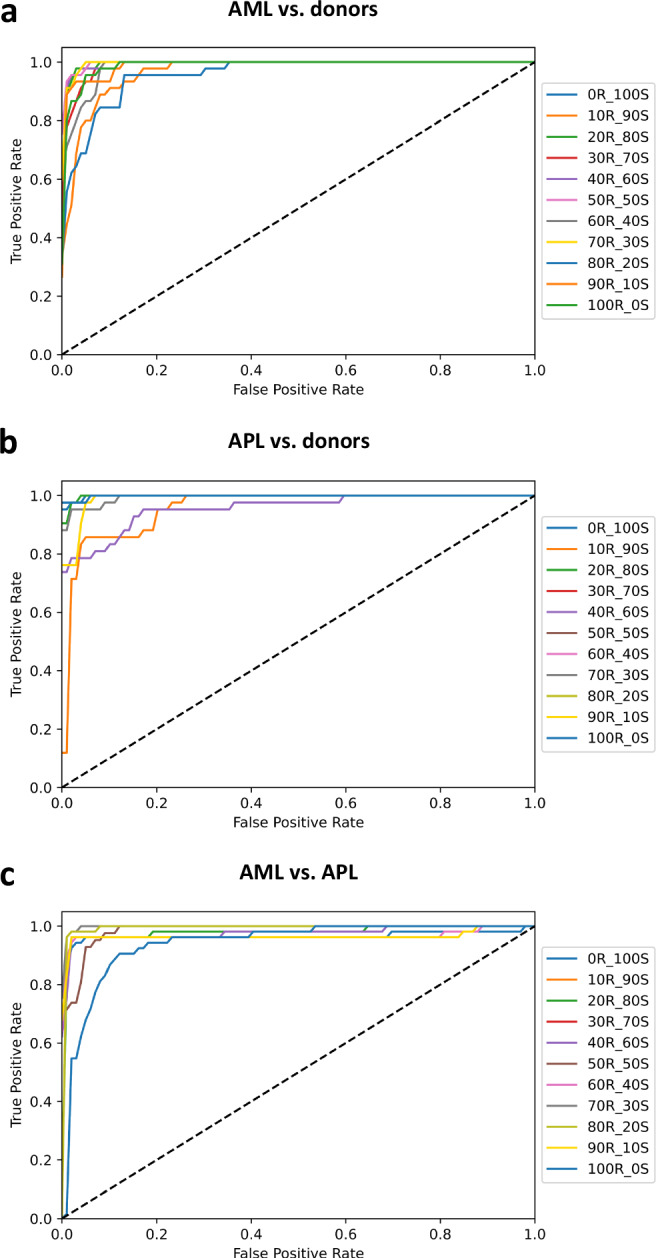
Proportional substitution of real data with synthetic data. Starting with real data only, we trained image classifiers to differentiate between AML vs. donors (**a**), APL vs. donors (**b**), and AML vs. APL (**c**). Then, we substituted real (abbreviated R) data with synthetic (abbreviated S) data incrementally in steps of 10% each. Notably, classification performance remained stable above an AUROC of 0.95, even when training on minimal or no real data and predominantly or fully synthetic training sets.

**Fig. 4:**
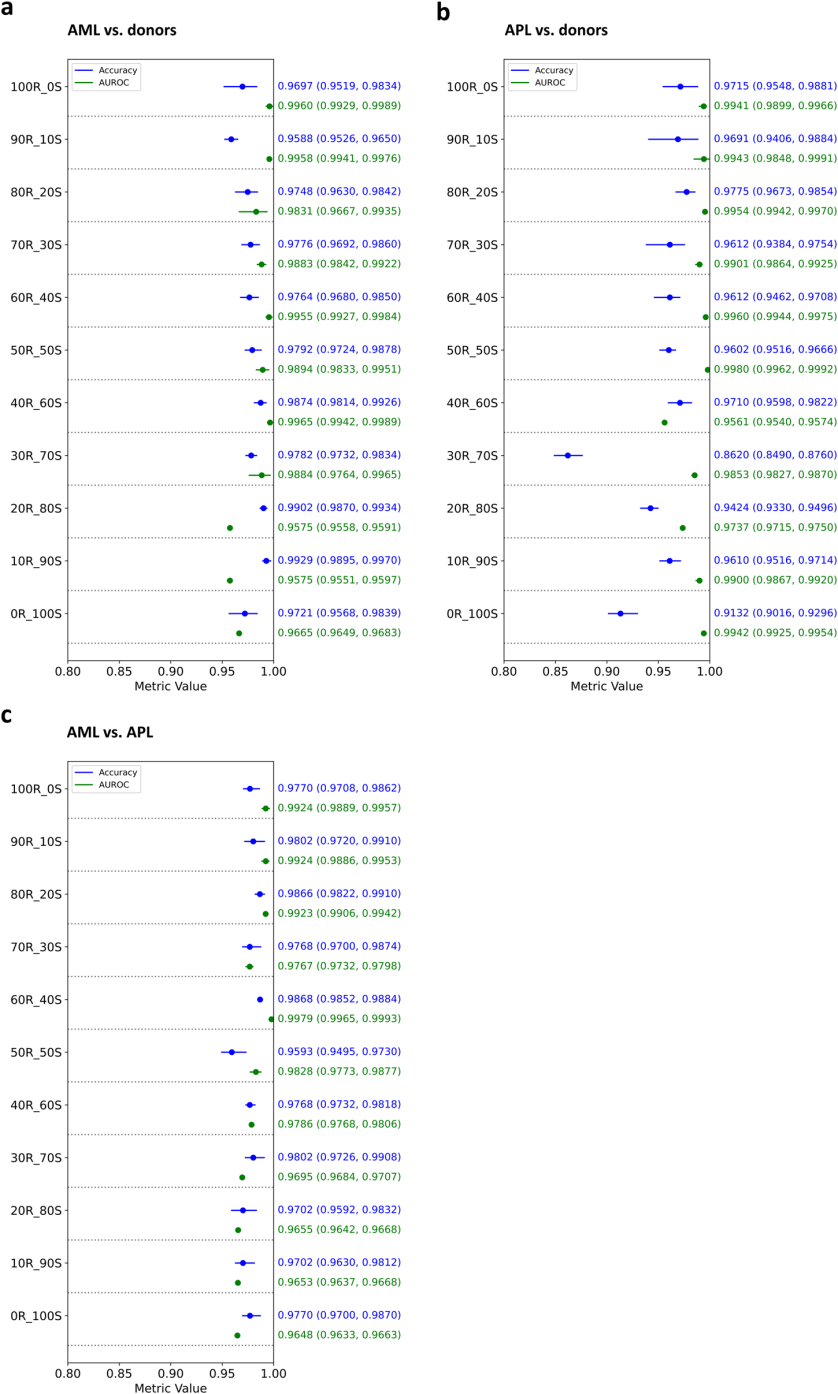
Forest plot demonstrating classification performance stability upon substitution of real data with synthetic data for classifier development. In line with this Figure, classification performance is measured by absolute values for accuracy and AUROC for the three use cases AML vs. donors (**a**), APL vs. donors (**b**), and AML vs. APL (**c**). Brackets show 95%-confidence intervals. Starting at 100% real (abbreviated R) data and gradually substituting real with synthetic (abbreviated S) data, classification performance remains stable above an AUROC of 0.95 for all three use cases, even with minimal real data or fully synthetic data.

After establishing high-quality synthetic images, we investigated how synthetic image data can augment or substitute the classification performance of pre-trained classifiers. In recent studies, we developed CNN-based classifiers for binary classifications of AML vs. stem cell donor samples^7^ as well as APL vs. non-APL-AML and APL vs. stem cell donor samples^8^ that achieved high individual classification performance. Since large patient cohorts are often difficult to obtain, we hypothesized that synthetic images could be used to compensate for lacking sample size. Baseline values for 100% real samples have been reported previously^7,8^. Using different CNN architectures, we were able to increase baseline metrics by further training on real images with fivefold cross-validation and an 80:20 train-test split (Table 1 and Figs. 3 and 4). We then iteratively substituted 10% of the real images in the training set with synthetic images until we reached 0% real and 100% synthetic training samples. Model performance was recorded at each step (Table 1 and Figs. 3 and 4) on a test set of real patients. Notably, across all three classification tasks (AML vs. donors, APL vs. donors, AML vs. APL), classifier performance, as measured by accuracy and AUROC, remained remarkably stable as the proportion of synthetic images increased, even to the point of training on a fully synthetic data set. For instance, even at 90% synthetic training data or 100% synthetic training data, the classifiers still achieved AUROC values above 0.95, indicating that synthetic images faithfully represent the variation in the original data and that the classification model could effectively generalize even with minimal or zero real training data. Conversely, we trained a model on 100% real data and tested it on only synthetic data, showing an accuracy of 97.15% and an AUROC of 0.9949 for AML vs. donors, accuracy of 94.22% and AUROC of 0.9903 for APL vs. donors, and accuracy of 95.44% and AUROC of 0.9803 for AML vs. APL (Supplementary Table 1).

**Table 1:** Classification performance at varying proportions of real and synthetic training data

Real [%]	Synthetic [%]	Accuracy [in %] (95%-CI)	AUROC (95%-CI)	Precision	Recall
**Proportion of real vs. synthetic samples**			**AML vs. donors**		
100	0	96.97 (95.19, 98.34)	0.996047 (0.9929, 0.9989)	0.99572	0.94331
90	10	95.88 (95.26, 96.50)	0.995847 (0.9941, 0.9976)	0.98780	0.98380
80	20	97.48 (96.30, 98.42)	0.983173 (0.9667, 0.9935)	0.97863	0.92712
70	30	97.76 (96.92, 98.60)	0.988340 (0.9842, 0.9922)	0.96761	0.96761
60	40	97.64 (96.80, 98.50)	0.995511 (0.9927, 0.9984)	0.99579	0.95951
50	50	97.92 (97.24, 98.78)	0.989438 (0.9833, 0.9951)	0.97983	0.98380
40	60	98.74 (98.14, 99.26)	0.996521 (0.9942, 0.9989)	0.99588	0.97975
30	70	97.82 (97.32, 98.34)	0.988440 (0.9764, 0.9965)	0.98312	0.94331
20	80	99.02 (98.70, 99.34)	0.957564 (0.9558, 0.9591)	0.97991	0.98785
10	90	99.29 (98.95, 99.70)	0.957564 (0.9551, 0.9597)	0.97309	0.87854
0	100	97.21 (95.68, 98.39)	0.966530 (0.9649, 0.9683)	0.99141	0.93522
			**APL vs. donors**		
100	0	97.150 (95.48, 98.81)	0.994108 (0.9899, 0.9966)	0.96610	0.93442
90	10	96.917 (94.06, 98.84)	0.994393 (0.9848, 0.9991)	0.9818	0.9642
80	20	97.758 (96.73, 98.54)	0.995414 (0.9942, 0.9970)	0.9818	0.9642
70	30	96.127 (93.84, 97.54)	0.990139 (0.9864, 0.9925)	0.98305	0.95081
60	40	96.127 (94.62, 97.08)	0.996076 (0.9944, 0.9975)	0.98305	0.95081
50	50	96.02 (95.16, 96.66)	0.998076 (0.9962, 0.9992)	0.98565	0.95752
40	60	97.10 (95.98, 98.22)	0.956109 (0.9540, 0.9574)	0.96774	0.98360
30	70	86.208 (84.90, 87.60)	0.985329 (0.9827, 0.9870)	0.96078	0.803278
20	80	94.240 (93.30, 94.96)	0.973781 (0.9715, 0.9750)	0.96610	0.93442
10	90	96.107 (95.16, 97.14)	0.990039 (0.9867, 0.9920)	0.98305	0.95081
0	100	91.32 (90.16, 92.96)	0.994257 (0.9925, 0.9954)	1.0	0.85245
			**AML vs. APL**		
100	0	97.70 (97.08, 98.62)	0.992412 (0.9889, 0.9957)	0.99186	0.97991
90	10	98.02 (97.20, 99.10)	0.992468 (0.9886, 0.9953)	0.99196	0.99196
80	20	98.66 (98.22, 99.10)	0.992376 (0.9906, 0.9942)	0.94339	0.94339
70	30	97.68 (97.00, 98.74)	0.976712 (0.9732, 0.9798)	0.99186	0.97991
60	40	98.68 (98.52, 98.84)	0.997933 (0.9965, 0.9993)	0.99595	0.98795
50	50	95.93 (94.95, 97.30)	0.982881 (0.9773, 0.9877)	0.98178	0.98013
40	60	97.68 (97.32, 98.18)	0.978686 (0.9768, 0.9806)	0.99595	0.98113
30	70	98.02 (97.26, 99.08)	0.969573 (0.9684, 0.9707)	0.98795	0.98795
20	80	97.02 (95.92, 98.32)	0.965589 (0.9642, 0.9668)	0.99180	0.97188
10	90	97.02 (96.30, 98.12)	0.965349 (0.9637, 0.9668)	0.97619	0.98795
0	100	97.70 (97.00, 98.70)	0.964851 (0.9633, 0.9663)	0.99186	0.97991

Next, we investigated whether model performance can be boosted by using all available real samples and adding variable amounts of synthetic samples. Again, a train-test split of 80:20 (fivefold cross-validation) was used. For AML vs. donors, we observed no increase in performance metrics, given that the baseline performance was already high without adding synthetic samples. For both use cases involving APL (APL vs. donors, APL vs. AML), we also found baseline performance to be high without adding synthetic samples, however, adding synthetic samples to the training set further increased accuracy from 94.17% (APL vs. donors, real samples only) to 97.09% (APL vs. donors, sample size doubled using synthetic samples) and 97.68% (AML vs. APL, real samples only) to 99.01% (AML vs. APL, sample size doubled using synthetic samples; Table 2).

**Table 2:** Classification performance for augmentation of real training data with synthetic images

No. of added synthetic images		AML vs. donors			
AML	donors	Accuracy [in %]	AUROC	Precision	Recall
0	0	94.881	0.98221	0.9557	0.9433
250	125	95.230	0.97182	0.9664	0.9752
500	250	95.904	0.99125	0.9875	0.9635
750	375	96.01	0.99878	0.9751	0.9354
1000	500	94.539	0.98336	0.9792	0.9554

		APL vs. donors			
APL	donors	Accuracy [in %]	AUROC	Precision	Recall
0	0	94.17	0.98412	0.9661	0.9344
125	125	95.54	0.992426	0.9559	0.9122
250	250	97.0297	0.995211	0.9333	0.9508
375	375	97.087	0.991495	0.9748	0.9211
500	500	97.087	0.99453	0.9333	0.9508

		AML vs. APL			
AML	APL	Accuracy [in %]	AUROC	Precision	Recall
0	0	97.68	0.992726	0.9918	0.9799
250	125	97.89	0.998411	0.9927	0.9889
500	250	98.344	0.995984	0.9959	0.9839
750	375	98.47	0.991128	0.9974	0.9821
1000	500	99.007	0.997121	0.9959	0.9839

## Discussion

Generative modeling has seen rapid advancements in recent years, leading to a multitude of applications enabled by open-access text-to-image models^9,10^. In clinical medicine, imaging is crucial in disease detection, highlighting the urgent need for publicly available data sets to train and validate models. Yet, data sharing is often hindered by privacy and regulatory concerns^11^. While GANs are frequently used in histopathology and radiology, where the main applications involve domain adaptation, data augmentation, and image-to-image translation^12,13^, studies in hematology are still sparse. In radiology, Prezja et al.^14^ used a similar setup to address data scarcity and sharing hurdles in X-ray imaging, training a GAN model to generate synthetic X-ray images of knee joints with varying degrees of osteoarthritis, which were then evaluated by medical experts in a visual Turing test, demonstrating the high quality of synthetic images and their usability for training image classification models. Further, Han et al.^15^ demonstrated that the generation of synthetic follow-up radiographs using a GAN and nearest neighbor embedding model boosted radiologists’ performance in detecting patients at risk of progressive osteoarthritis.

In hematology, the microscopy of peripheral blood or bone marrow has been a diagnostic cornerstone for decades^16,17^. Several pioneering studies focused on cell-level image generation. Barrera et al.^18^ proposed SyntheticCellGAN, a framework for generating synthetic white blood cell images from peripheral blood smears, including lymphocytes, monocytes, eosinophils, neutrophils, basophils, atypical promyelocytes, and hairy cells. They first trained a classifier on real samples and then used it to predict classes of synthetic samples. Second, they trained a classifier on synthetic samples and predicted classes of real samples, reporting accuracies between 87–100% per cell class^18^. Correspondingly, Liu et al.^19^ employed generative modeling to cell-level image synthesis of neutrophils, eosinophils, monocytes, and lymphocytes using a combinatorial approach with variational autoencoders and StyleGAN. Using bone marrow cell images, Hazra et al.^20^ propose C-WGAN-GP (classifier-Wasserstein-GAN with gradient penalty) with a sequential CNN in order to classify 12 different cell types and report performance gains for the addition of synthetic cell images. In a subsequent study, the same authors report improved cell-level image generation for their proposed method compared to other GAN architectures, achieving similar classification accuracies when using CNNs on real and their synthetic cell images^21^.

While previous studies have focused on cell-level image generation, our study targets entire ROIs with diagnosis-level labels and an end-to-end diagnosis classification task. Using a pre-trained StyleGAN-2-Ada^22^, we generated high quality images that withstood evaluation by experienced hematologists in a visual Turing test. Upon closer inspection, recurring structural similarities in cell arrangement or unnatural disparities in chromatin density as a cue to identify synthetic images could pertain to limitations in data generation with a limited sample size and, potentially, data diversity. Increasing both sample size and diversity may help the model to learn more representative features especially pertaining to chromatin structure, further increasing the degree of interchangeability of synthetic and real data as well as generalizability. In subsequent disease detection with varying proportions of real and synthetic images, we observed model performance to remain remarkably stable as the percentage of real images in the training set decreased and the percentage of synthetic images increased. Even using a fully synthetic training set, model performance exceeded an AUROC of 0.95 for all use cases, demonstrating the effectiveness of synthetic images for model training and evaluation. To this end, we provide full open access to our synthetic data for the scientific community to enable external model development and performance assessment.

From a clinician’s perspective, a training data set of >1200 AML samples may be considered large, however, deep learning in general thrives on usually much larger data sets. This poses a significant limitation for sample size augmentation with GANs, as sufficiently large data sets are needed to effectively train a generative model. This creates a paradox: improving classification performance requires more data, which GANs can generate, but initially training GANs requires a sufficiently large sample size. While model selection in this study was based on loss functions, incorporating Fréchet Inception Distance could provide an additional criterion for identifying the optimal epoch for generating high-quality synthetic images and mitigating potential overfitting. Another limitation is data diversity in smaller data sets. Given the rarity of AML and APL, our dataset includes cases collected over the past two decades, encompassing both recently prepared and older bone marrow smears. As such, variability in staining intensity, particularly for red blood cells (RBCs), and occasional signs of neutrophilic disintegration may reflect differences in sample age, potentially affecting learned features during image generation. Further, an absence of megakaryocytes was noticed in synthetic donor images, potentially reflecting the tendency of the generative model to underrepresent sparse features that appear infrequently in the training set. The latter may be a limitation of a patch-based approach, as megakaryocytes occupy a larger image area and may therefore be underrepresented in ROIs. Implicit patient-specific morphologies may influence the generation of synthetic samples that will ultimately lack diversity and therefore deviate from the actual representation of the population the sample is drawn from. Hence, the generation of synthetic samples still relies on large and diverse data sets if the desired synthetic samples are to fully represent the underlying patient population. While latent space perturbations offer the possibility to artificially increase the representation of distinct image properties potentially pertaining to specific morphologies, sufficient sample size and diversity to represent these features is still key, either to enhance model training for rare diseases with distinct features such as APL, or to generate samples rich in certain morphologies for educational purposes. Our AML training sample stems from multiple previous multicenter clinical trials conducted in Germany, predominantly involving adult Caucasian patients who were fit enough to receive intensive treatment. Thus, the generalizability of our synthetic sample to other patient populations may vary. Further, a lack of computational resources in facilities striving to generate synthetic data may pose another constraint on widespread accessibility to the technology. Potentially, cloud computing solutions may help overcome this issue, however, data privacy should be maintained when using commercial decentralized resources.

Due to stringent privacy regulations and institutional data sharing constraints, access to large and diverse data sets is often limited, particularly in rare diseases in hematology. Synthetic images, which only imitate features represented in a patient population rather than copying real samples, offer a privacy-compliant alternative that can be shared among institutions, enabling collaborative development of diagnostic models and serving as benchmark data sets fostering validation and translation of algorithms into clinical practice. While we demonstrate that synthetic data can aid in the training and evaluation of image-based classifiers, further studies are needed to generate and evaluate multimodal data linking images to synthetic patient histories, genetics, and treatment responses. Such fully synthetic digital avatars could enable synthetic clinical trial cohorts, alleviating the burden of enrolling ever-larger control cohorts in an era of precision medicine that is facing increasingly costly and time-consuming clinical trials.

In conclusion, we demonstrate the feasibility of GANs to generate high-quality BMS images that can withstand the watchful eyes of experienced hematologists tasked with distinguishing real from synthetic samples. Classification performance at varying proportions of real and synthetic samples for AML and APL detection shows that synthetic images are a viable substitute for real images and that training image classifiers is feasible with low proportions of real data or using fully synthetic training sets. These synthetic images can be generated at will and may help overcome data scarcity issues and privacy concerns.

## Methods

### Study cohorts and bone marrow smear digitization

**Fig. 5:**
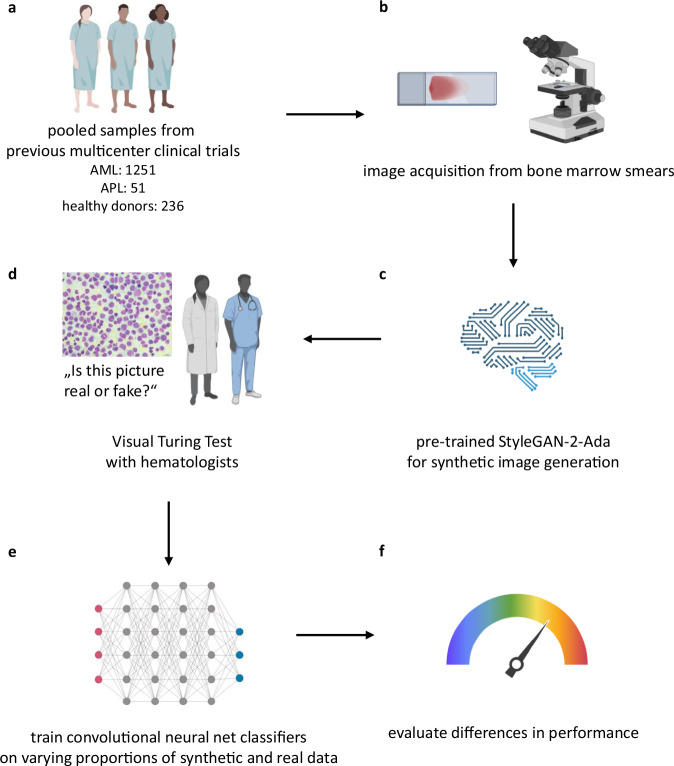
Workflow for synthetic image generation and evaluation. Samples were obtained from previous multicenter clinical trials (**a**). Bone marrow smears (BMS) were digitized using the Nikon Eclipse E600 microscope with the Nikon DS-Fi2 mounted camera and Nikon Imaging Software Elements D4 to obtain high resolution images (2560 × 1920 pixels) of representative areas at 40-fold magnification (**b**). StyleGAN-2-Ada was pre-trained on Flickr-Faces-HQ and transferred to our image data in order to generate synthetic BMS images (**c**). After convergence, synthetic images were presented to hematologists using a web-based tool in order to judge each image as either real or synthetic (**d**). After this proof-of-concept, both real and synthetic images were fed to convolutional neural network classifiers in varying proportions (**e**). Finally, performance for each percentage-wise combination of real and synthetic data was evaluated (**f**). This figure was partially created using Biorender.

Bone marrow smears from 1251 adult AML patients (Table 3 and Fig. 5a) were retrieved from previously reported multicenter trials (AML96 [NCT00180115]^23^, AML2003 [NCT00180102]^24^, AML60+ [NCT00180167]^25^, and SORAML [NCT00893373]^26^) and the German Study Alliance Leukemia (SAL) registry (NCT03188874), spanning patients treated between 1996 and 2023. Eligibility criteria for these trials can be consulted under the provided references. Only bone marrow smears from initial diagnosis from adult patients with validated diagnosis of AML at a blast cut-off of 20% were used for the purpose of this study (which may not include patients with genetically-driven definitions of AML irrespective of blast count or AML/MDS overlap). Additionally, bone marrow smears from 51 adult acute promyelocytic leukemia (APL, AML-M3) patients were included as a separate cohort (Table 3). The control cohort (Table 3) was composed of 236 adult individuals who donated bone marrow at our center, as previously reported^27^. All stem cell donors underwent rigorous medical evaluation prior to stem cell donation, ruling out any disorders potentially disqualifying them to undergo donation. Still, variations in cellular composition in donor smears may occur due to secondary causes unrelated to hematologic malignancies. In the context of stem cell donation, such shifts could be attributable to granulocyte-colony stimulating factor (G-CSF) administration, which is routinely used to mobilize stem cells prior to collection. All participants gave their written informed consent according to the Declaration of Helsinki^28^. All experiments were previously approved by the Institutional Review Board of the TUD Dresden University of Technology (EK 98032010).

**Table 3:** Baseline patient and donor characteristics

Parameter	non-M3 AML	APL (AML-M3)	Bone marrow donors
*N*	1251	51	236
Age, median (IQR)	57 (38–67)	50.5 (42.25–58)	31 (25–39)
Sex, *n* (%)			
Female	583 (46.6)	28 (54.3)	71 (30)
Male	668 (53.4)	23 (45.7)	165 (70)
ELN2017 risk, *n* (%)			
Favorable	380 (30.4)	–	–
Intermediate	521 (41.6)	–	–
Adverse	249 (19.9)	–	–
Missing	101 (8.1)		
Sanz-Score, *n* (%)			
Low	–	8 (15.7)	–
Intermediate	–	10 (19.6)	–
High	–	6 (11.8)	–
Missing	–	27 (52.9)	–
AML type, *n* (%)			
De novo	969 (77.5)	46 (90.5)	–
sAML	177 (14.1)	0	–
tAML	101 (8.1)	5 (9.5)	–
Missing	4 (0.3)	0	
Laboratory			
WBC in GPt/L, median (IQR)	17.8 (3.2–52.5)	1.3 (0.7–6.4)	–
Hb in mmol/L, median (IQR)	6.0 (5.1–7.1)	6.3 (5.3–6.9)	–
Plt in GPt/L, median (IQR)	64 (38–103)	27 (18–57)	–
% PB blasts, median (IQR)	23.5 (4–60)	14.75 (1.3–63)	–
% BM blasts, median (IQR)	60.5 (40–80)	63 (53.5–76)	–

*BM* bone marrow, *ELN2017* European Leukemia Net 2017, *Hb* hemoglobin, *IQR* interquartile range, *n/N* number, *PB* peripheral blood, *Plt* platelet count, *sAML* secondary AML, *tAML* therapy-related AML, *WBC* white blood cell count.

Bone marrow smears were manually stained using the May-Grünwald-Giemsa method^16^. Evaluable regions-of-interest (ROI) were manually photographed using a Nikon Eclipse E600 microscope with a Nikon DS-Fi2 mounted camera and Nikon Imaging Software Elements D4 to obtain high-resolution image data (2560 × 1920 pixels) (Fig. 5b). ROIs were selected based on representative and evaluable smear image areas, focus, staining adequacy, and technical image quality. No specific criteria were applied regarding the presence or absence of particular cell types, ensuring a diverse representation of cellular morphology reflective of real-world diagnostic variability. Only one ROI was captured per patient. Each ROI corresponded to an area of 171 × 128 µm.

### Generative adversarial networks and visual Turing test

Working with a limited number of training samples for generative modeling poses a significant risk of the model capturing noise rather than useful patterns, leading to divergent training and overfitting. Given the limited training data in our setup, we employed a GAN architecture designed to handle small data sets effectively. Karras et al.^29^ proposed StyleGAN2-Ada, a model architecture incorporating adaptive discriminator augmentation. This method stabilizes model training by augmenting real training data with transformed copies. A different transformation is used for each copy, which encourages the discriminator to learn more robust features that are less sensitive to minor variations. For model input, rectangular images (2560 × 1920 pixels) were re-sized to 1024 × 1024 pixels to meet the required StyleGAN2-Ada input specifications, ensuring compatibility with the model’s architecture. To initialize features for shape and color generation, StyleGAN2-Ada was pre-trained on Flickr-Faces-HQ^22^, a high-quality diverse data set of 70,000 images at 1024 × 1024 pixels. The pre-trained model was used to generate bone marrow smear images based on our training data (Fig. 5c). Hyperparameter search and optimization was performed using the Optuna^30^ framework. Models were trained at the High Performance Computing (HPC) Facility at TUD Dresden University of Technology using eight NVIDIA A100 GPUs. The models were built in Python version 3.7.10 (Python Software Foundation, DE, USA, 2023) as the base of our environment. Additional key packages include PyTorch (ver. 1.13.0), TorchVision (ver. 0.14.0) and TorchAudio (ver. 0.13.0), all configured with CUDA (ver. 11.6) to leverage GPU acceleration. To ensure consistency with the pre-trained model used for feature extraction, all images were normalized using ImageNet statistics by centering pixel values based on the dataset’s mean and scaling by its standard deviation. This standard normalization approach aligns the input distribution with the original training data, improving feature compatibility and stability in similarity assessments. To assess the quality of the synthetic images generated by GANs, we employed two quantitative metrics: Euclidean distance similarity score^14^ and Structural Similarity Index Measure (SSIM)^31^. Euclidean distance varies inversely with similarity: A lower Euclidean distance indicates greater similarity between a real and synthetic image pair, whereas a higher Euclidean distance reflects greater dissimilarity. SSIM has a range from 0 to 1, with a value of 1 indicating perfect similarity between a given synthetic and real image pair. To calculate the Euclidean distance similarity scores, the InceptionV3 model was used to extract feature vectors for both the synthetic and real image data sets for all real-synthetic image pairs. This is followed by calculation of the Euclidean distance for all possible synthetic-real pairs. Importantly, the exact InceptionV3 pipeline was implemented before calculating similarity scores. In parallel, the SSIM metric was implemented, which focuses on the perceptual quality of the images, taking into account luminance, contrast and structure, instead of comparing raw pixel intensities. To further assess the perceptual quality and realism of the generated images, we conducted a visual Turing test^32^ with experienced hematologists who were tasked with evaluating whether a given image originated from the real data set or was synthetically generated (in Germany, hematology and medical oncology are a joint specialty, i.e. hematologists are also medical oncologists, and medical oncologists are also hematologists). If the generated images convincingly resembled real microscopic images, observers would find it challenging to differentiate them. To this end, a web application was created that contained a data set of both real and synthetic BMS images, which were shuffled and randomly presented one-by-one to the user. All images were re-sized to the same resolution and size (1024 × 1024 pixels) in order not to give away an image’s label (Fig. 5d). Users were tasked with making a global image-based judgment, labeling each image as either “real” or “synthetic”. A more detailed morphological assessment or differential counts were not conducted. The task was intentionally unstructured to mimic real-world decision-making conditions and avoid guiding users toward specific cues. Users were able to devote as much time as needed for inspection of a single image, however, after assigning a label they could not revisit the same image and change their decision in hindsight. Upon completion, the user was presented with his/her individual accuracy as well as a comparison to other users.

### Convolutional neural network classifier and classification performance analysis

To evaluate the feasibility of training and testing computer vision models with synthetic data, we iteratively trained and tested CNN-based classifiers using varying proportions of real and synthetic data. We did not exclude or selectively curate any synthetic images. All generated images were used for classification. To demonstrate interchangeability of high-quality synthetic and real data, classifier training was performed by starting off with only real data for the first iteration of training. Subsequently, increasing proportions of real data were randomly removed (in steps of 10% for each of the cohorts) and were substituted with correspondingly sized numbers of synthetic images. Classifiers were re-trained at every increment, and performance was measured in order to evaluate performance shifts at varying proportions of real and synthetic images in the cohorts. After establishing the performance for substituting real images with synthetic images, in a follow-up experiment, we augmented the cohorts by adding synthetic images on top of the real images in the training sets and measured performance gains (if any) at increasing sample sizes. Image-level labels (“AML”, “APL”, “donor”) were used to train classifiers end-to-end, as previously described in detail^7,8^. Image-level labels were derived from standard clinical workflows and were based on the results and reports of cytomorphology, histopathology, flow cytometry, cytogenetics, molecular genetics, and clinical assessment. Importantly, no manual labeling of cells on images was performed, rather images were labeled as a whole. Only one ROI image per patient was used, ensuring that image data from the same patient could not be present in training and test set at the same time. An 80:20 split between training and validation sets was used along with fivefold cross-validation, with the validation set strictly excluded from training to ensure that the model’s performance is evaluated on unseen data. Image augmentation techniques such as rotating images, mirroring, linear transformations, color shifts, and brightness adjustments were applied to accommodate for different sample sizes between classes. Again, the Optuna^30^ framework was used for hyperparameter optimization. Performance was evaluated using the area under the curve (AUC) of the receiver operating characteristic (ROC) in conjunction with precision and recall. Precision (positive predictive value) is the proportion of true positives among all positive predictions. Recall (sensitivity) is the proportion of correct positive predictions among all truly positive elements (in accordance with the ground truth) (Fig. 5e, f).

## Supplementary information

Supplementary Information

## Data Availability

The data supporting the conclusions of this article is available under 10.6084/m9.figshare.28449269.

## Code availability

The synthetic data generation pipeline is available via GitHub under https://github.com/wangzizhe/gan-framework.
