# Supplementary material for: Synthetic bone marrow images augment real samples in developing acute myeloid leukemia microscopy classification models
Source: NPJ Digit Med. 2025 Mar 22;8:173. doi: 10.1038/s41746-025-01563-9 (PMC11928482; doi:10.1038/s41746-025-01563-9)
Supplement: Supplementary file 1 — Supplementary Information [file 41746_2025_1563_MOESM1_ESM.docx]

**Supplemental Figures**

**
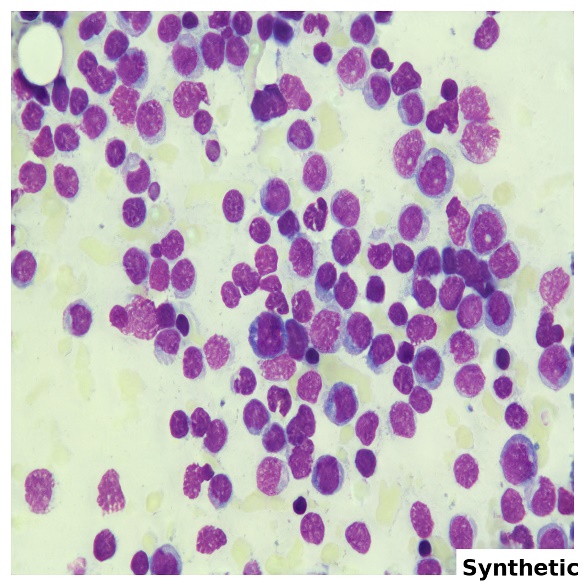

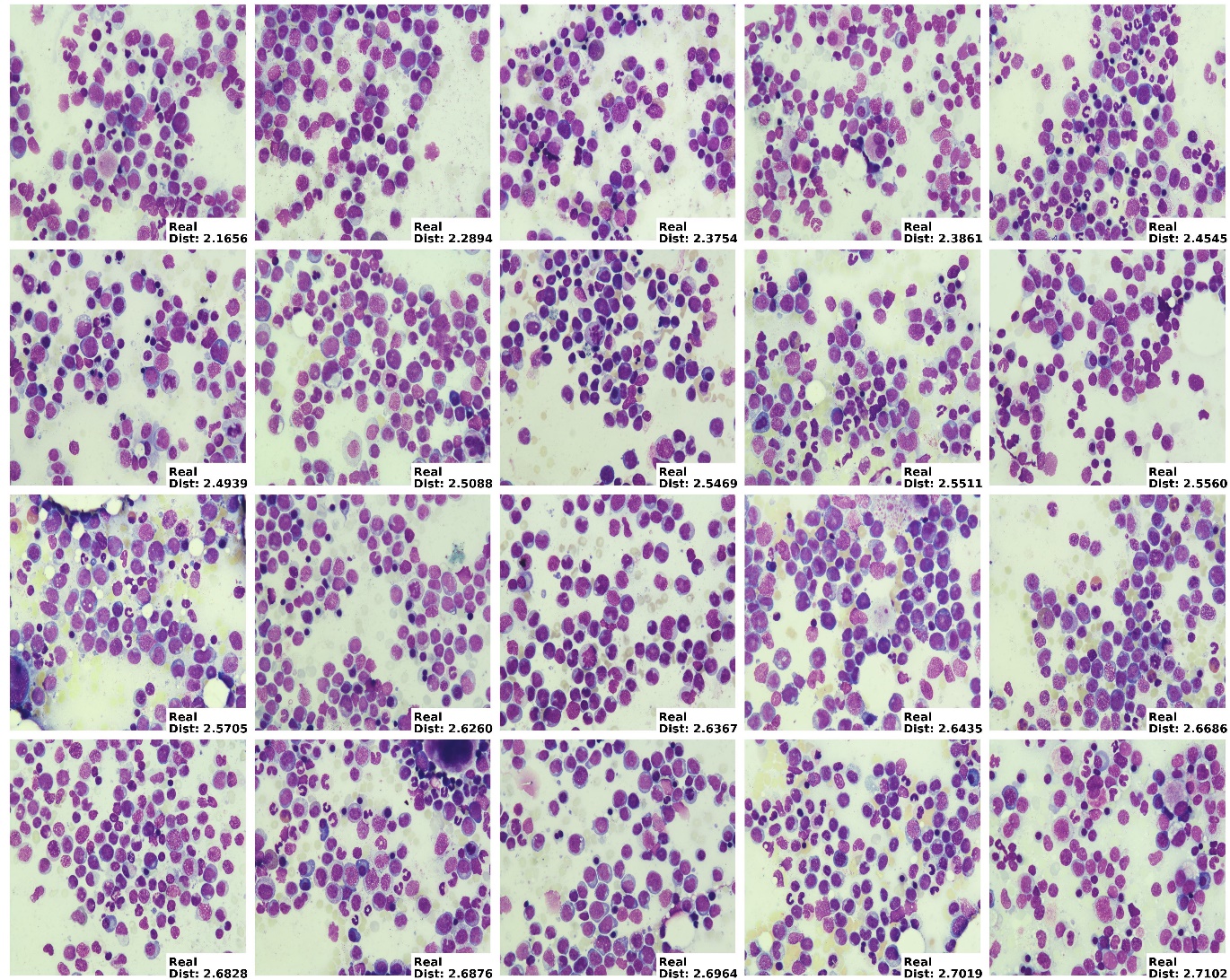
**

**Supplemental Figure 1. Euclidean distance similarity scores for synthetic-real AML image pairs.** Synthetic AML image compared to the 20 paired real AML images that had the lowest Euclidean distance similarity scores, indicating sufficient similarities between real-synthetic image pairs while avoiding direct replication. Numerical score values are given per sample in the lower right corner of each panel.

**
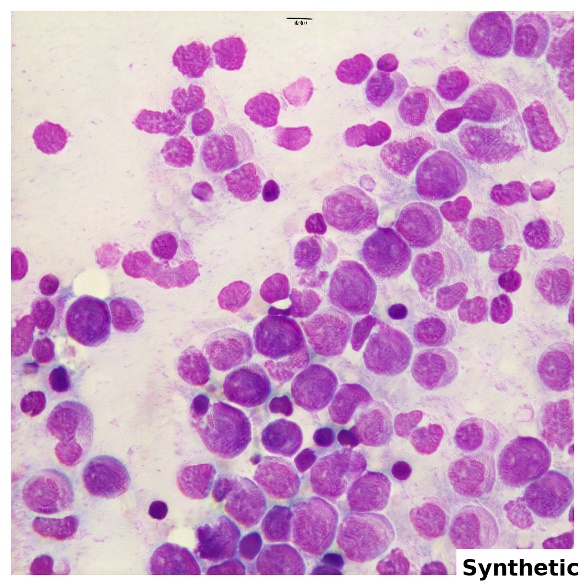

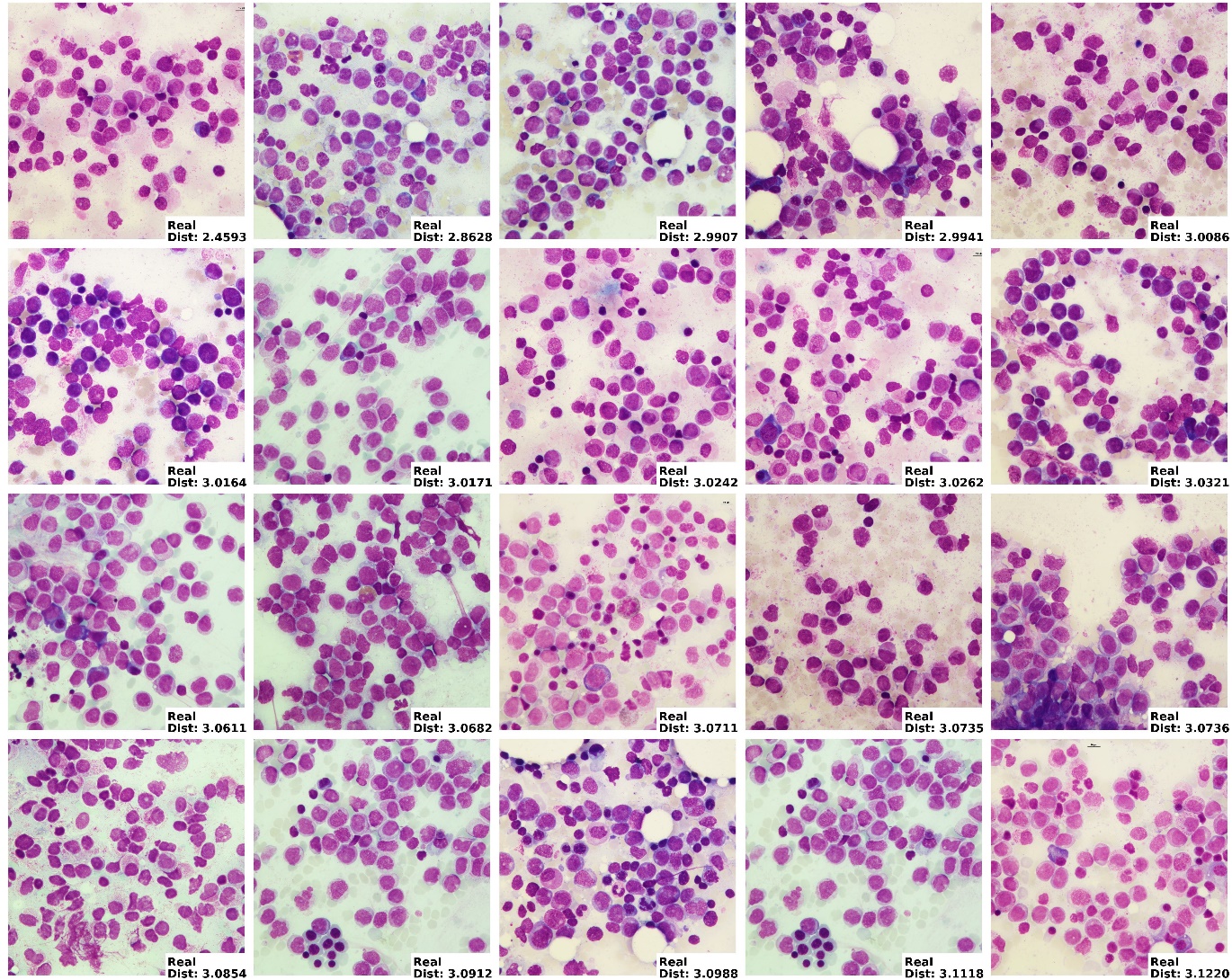
**

**Supplemental Figure 2. Euclidean distance similarity scores for synthetic-real APL image pairs.** Synthetic APL image compared to the 20 paired real APL images that had the lowest Euclidean distance similarity scores, indicating sufficient similarities between real-synthetic image pairs while avoiding direct replication. Numerical score values are given per sample in the lower right corner of each panel.


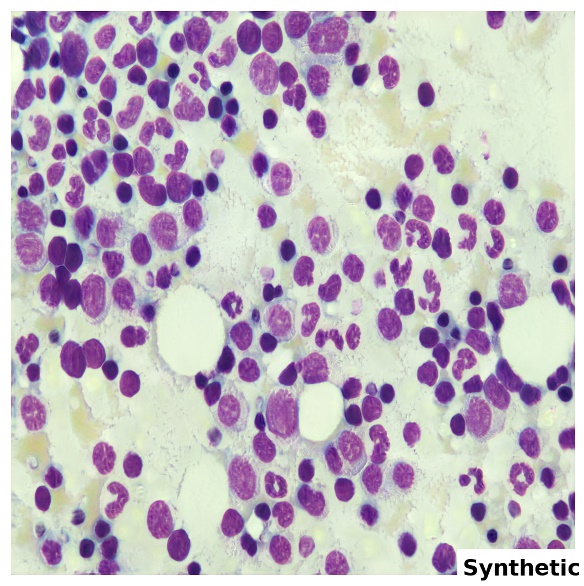

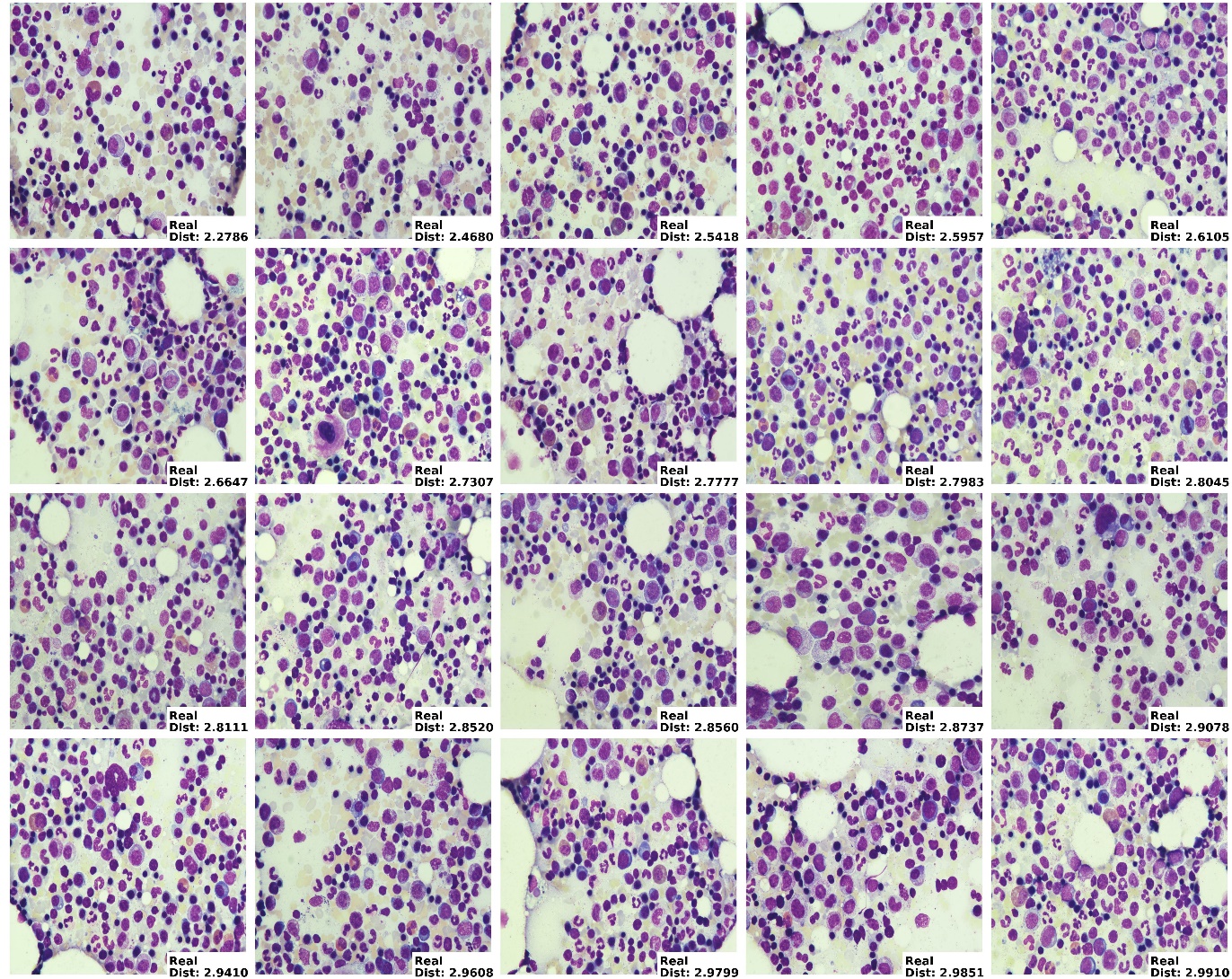


**Supplemental Figure 3. Euclidean distance similarity scores for synthetic-real donor image pairs.** Synthetic donor image compared to the 20 paired real donor images that had the lowest Euclidean distance similarity scores, indicating sufficient similarities between real-synthetic image pairs while avoiding direct replication. Numerical score values are given per sample in the lower right corner of each panel.


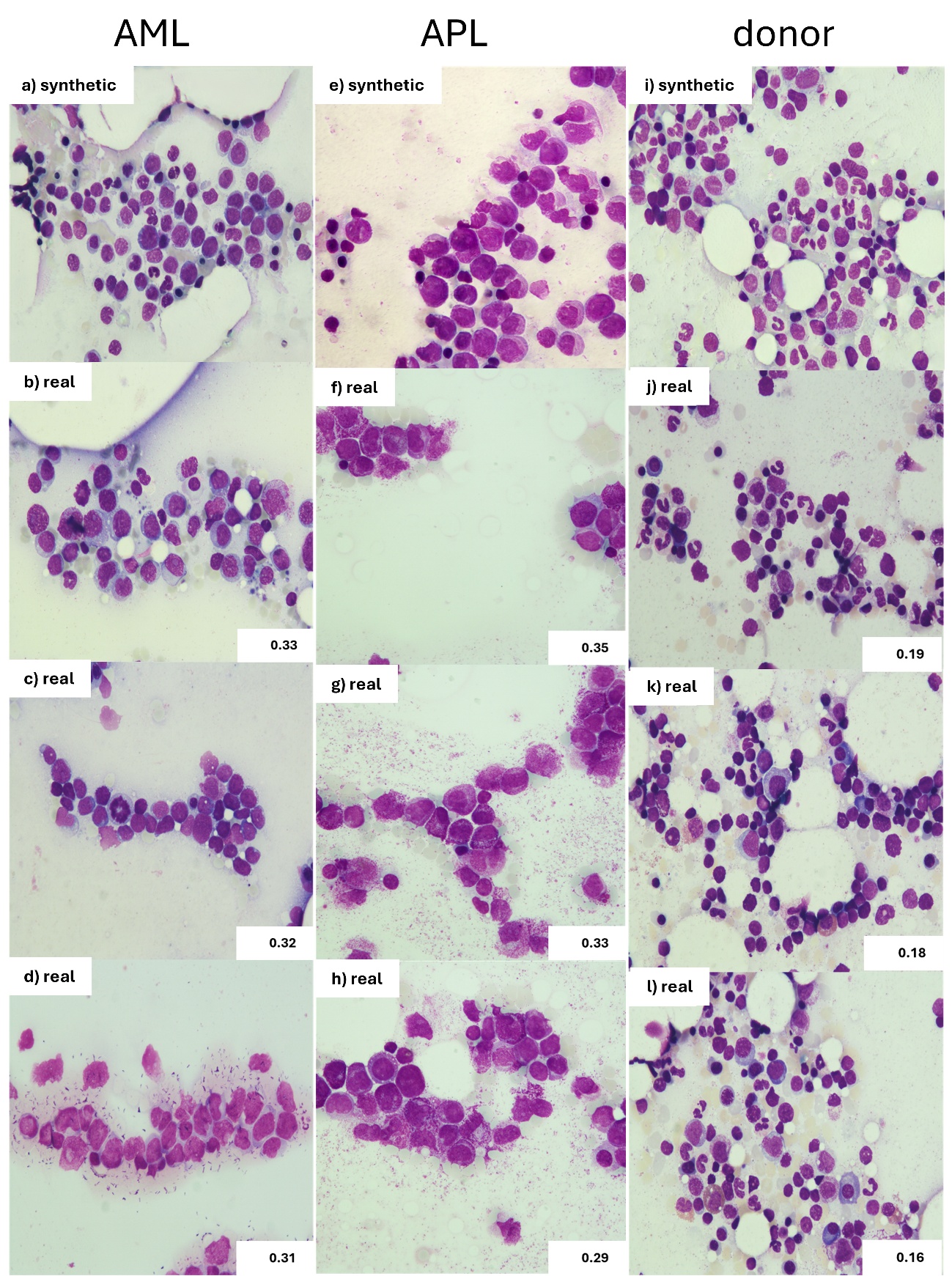


**Supplemental Figure 4. Structural Similarity Index Measure scores.** The top row (a, e, i) show examples of synthetic images for AML (a), APL (e), and donor (i) samples. Below are paired real images with the top three highest corresponding structural similarity index measure scores for the respective synthetic sample for AML (b, c, d), APL (f, g, h), and donors (j, k, i). Numerical score values are given per sample in the lower right corner of each panel. These indicate low structural similarity highlighting adequate abstraction in the image generation process.

**Supplemental Tables**

**Supplemental Table 1. Classification performance for training on 100% real data and testing on synthetic data**

|  |  | **Accuracy [in %]**  **(95%-CI)** | **AUROC (95%-CI)** | **Precision** | **Recall** |
| --- | --- | --- | --- | --- | --- |
| AML vs. donors | | 97.15 (95.179, 98.932) | 0.9949 (0.9890, 0.9979) | 0.9894 | 0.9451 |
| APL vs. donors | | 94.22 (92.418, 97.487) | 0.9903 (0.9839, 0.9953) | 0.9647 | 0.9559 |
| AML vs. APL | | 95.44 (92.260, 97.881) | 0.9803 (0.9680, 0.9942) | 0.98199 | 0.9836 |
